# The thorax musculature of Anisoptera (*Insecta:* Odonata) nymphs and its evolutionary relevance

**DOI:** 10.1186/1471-2148-13-237

**Published:** 2013-11-01

**Authors:** Sebastian Büsse, Thomas Hörnschemeyer

**Affiliations:** 1 Department of Morphology, Systematic & Evolutionary Biology, J-F-Blumenbach Institute for Zoology & Anthropology, Georg-August-University Göttingen, Göttingen, Germany

**Keywords:** Dragonflies, Homology with Neoptera (Insecta), Odonata nymphs, Musculature, Insect flight apparatus, Homologization scheme

## Abstract

**Background:**

Among the winged insects (Pterygota) the Odonata (dragon- and damselflies) are special for several reasons. They are strictly aerial predators showing remarkable flight abilities and their thorax morphology differs significantly from that of other Pterygota in terms of the arrangement and number of muscles. Even within one individual the musculature is significantly different between the nymphal and adult stage.

**Results:**

Here we present a comparative morphological investigation of the thoracic musculature of dragonfly (Anisoptera) nymphs. We investigated representatives of the Libellulidae, Aeshnidae and Cordulegasteridae and found 71 muscles: 19 muscles in the prothorax, 26 in the mesothorax and 27 in the metathorax. Nine of these muscles were previously unknown in Odonata, and for seven muscles no homologous muscles could be identified in the neopteran thorax.

**Conclusion:**

Our results support and extend the homology hypotheses for the thoracic musculatures of Odonata and Neoptera, thus supplementing our understanding of the evolution of Pterygota and providing additional characters for phylogenetic analyses comprising all subgroups of Pterygota.

## Background

The evolutionary diversification of highly functional structures can elucidate the adaptation of organisms to special habitats and/or living conditions. The mostly direct flight muscle system of the Odonata e.g. [1-4] facilitates impressive flight skills, enabling them to be the aerial key predators among the insects [5].

Aside from the Holometabola, Odonata is the insect group with the greatest variation between the habitat preferences of the nymphs and the adult. The adult is an elegant and agile aerial predator whereas the predaceous nymphs are aquatic e.g. [5-7]. The ontogenetic development of the thoracic morphology of Odonata has not been extensively studied. Poletaïev [8] reported that the wing buds occur at the 3^rd^ or 4^th^ instar but that the corresponding musculature is still undiscernible Maloeuf [9] remarked that the flight muscles in those instars are diminutive. He also stated that the Odonata nymphs have larger and a higher number of leg and cervical muscles than the adults. The amount and the kind of muscles differ significantly between nymphal and adult Odonata e.g. [1,9]. During the ontogenesis the thoracic muscles are, in part, newly formed, transformed or reduced [9,10]. The extent of these modifications seems to be exceptional in the Odonata compared to other non-holometabolous Pterygota, which mostly exhibit a complete set of muscles from the first instar onward e.g. [11,12].

Generally, Odonata can be divided into three groups: The well-known dragonflies (Anisoptera) and damselflies (Zygoptera) and the not commonly known and enigmatic *Epiophlebia.* Recent phylogenetic studies of these three groups of Odonata based on morphological as well as molecular data, supported a sister group relationship of Zygoptera and Epiprocta, which comprises Anisoptera and *Epiophlebia*[13-19]. In a single study *Epiophlebia* is designated the sister group of the Cordulegastridae (Anisoptera) [20]. The thoracic musculature of adult Anisoptera e.g. [2,3,21] and also of *Epiophlebia superstes* Sélys, 1889 [1] is comparatively well-investigated. The pterothorax of adult Zygoptera has been studied comprehensively by Büsse et al. [4] and to some degree also by Asahina [1] and Ninomiya & Yoshizawa [22] whereas the thoracic musculature of Odonata nymphs has received little attention so far [1,9,10]. We therefore focus our study on the latter topic to fill a gap of knowledge and help to elucidate the ground pattern of the odonatan thorax.

Insect flight is often considered as a key factor for the evolutionary success of the Pterygota [23]. However, the origin and evolutionary development of the insect flight apparatus are not well understood. The motion transmission from the flight musculature to the wings is implemented in two different ways. The muscles and wing base sclerites in Odonata form a direct flight mechanism. Dorso-ventral muscles are attached directly to elements of the wing base,directly actuating the wings. Nevertheless, Pfau [3] and Büsse et al. [4] showed that Odonata also have a few indirect muscles that participate in the wing beat.

In all other Pterygota the indirect flight mechanism dominates the movement of the wings. Strong dorso-longitudinal muscles that are very small or missing in Odonata, deform the thorax. This deformation moves the wings via the wing base sclerites e.g. [7,24]. These differences in the flight mechanisms indicate that the thorax of Odonata is a highly derived character system. This, however, makes identifying the homologies of sclerites and muscles between Odonata and the remaining Pterygota difficult, and a topic of ongoing discussion e.g. [4,22,25]. The present investigation supplements this with a hitherto largely neglected character set.

The relationships between the Odonata, Ephemeroptera and the Neoptera are still under discussion [26-29]. All three possible combinations of these three taxa have been discussed: The sistergroup relationship between Ephemeroptera and Odonata, i.e. the Palaeoptera hypothesis, was mentioned by Martynov [30]. He divided the Pterygota into two groups: the Palaeoptera, or “old winged” insects and the Neoptera, or “new winged” insects. This grouping is based mainly on the inability of the groups comprised by Palaeoptera to fold their wings above the abdomen and the similarity of the wing base sclerites [30-39]. In addition, DNA [27,40] and recent morphology-based analyses [28,29,41] also support the Palaeoptera hypothesis. The second hypothesis proposes Odonata and Neoptera as sistergroups under the name Metapterygota. This hypothesis is supported by apomorphies including the lack of the ecdysis in the winged stage, the number and position of the mandible articulations and the corresponding loss of several muscles, etc. [12,23,42-50]. The DNA analysis of Ogden and Whiting [26] also supports the Metapterygota hypothesis. However, the latter study used a method called direct optimization, which, according to more recent studies [51-53] is an unreliable procedure.

The third scenario, the Chiastomyaria hypothesis, proposes a sistergroup relationship of Ephemeroptera and Neoptera [54-56]. The strong dorso-longitudinal indirect wing depressor often considered as symplesiomorphic for Pterygota, and the direct sperm transfer of the male to the female gonopore, which was often considered as convergent, are interpreted as apomorphies for this grouping. Some molecular-based studies [57,58] also support this hypothesis, which generally is seen as the most improbable of the three [49]. However, a better understanding of these relationships is indispensable for elucidating the ground pattern of Pterygota and the understanding of the evolution of insect flight [59].

One key factor to reconstructing the basal phylogeny of Pterygota and to the origin and evolution of insect flight is the flight apparatus of Odonata. To be able to utilize its abundance of characters for phylogenetic analyses, the thoracic musculature of Odonata must be convincingly homologized with the musculature of Neoptera.

Presently there seems to be widespread agreement on a ground pattern hypothesis for the wing base sclerites and for the flight musculature in Neoptera [12,60-62]. Even homologies between Ephemeroptera and Neoptera are mainly resolved [61,63], while hypotheses on the homologies between Odonata and the remaining Pterygota are still under discussion [3,4,22,61,63].

In this comparative morphological analysis we study the musculature of the thorax of late nymphal instars from three groups of Anisoptera. A homologization scheme for the thorax musculature with a generalized neopteran thorax [62], following and supplementing the study of Büsse et al. [4], is presented. Our study allows for new insights into the evolution of the odonatan thorax and may help to elucidate the early evolution of the insect flight apparatus.

## Results

Throughout this work the attachment points of the muscles are named such that the non-functional or non-moving end is called the point of origin and the functional or moving end is the point of insertion. The muscle names are formed accordingly. In a few cases this leads to differences in the muscle descriptions in comparison to other authors e.g. [1,9,62] even though the same muscle is addressed.

In the thorax of anisopteran nymphs a total of 71 muscles were found: 19 muscles in the prothorax, 26 in the mesothorax and 27 in the metathorax. Nine of these muscles were previously unknown for Odonata: Ispm1, IIscm1 (IIIscm1), IIscm2 (IIIscm2), IIscm8, IItpm3 (IIItpm3) and IIIscm4 and seven muscles (Ipcm9, Itpm7 – Itpm11, IIscm8) have no homologue in the neopteran thorax.

The musculature of the thorax of *Sympetrum vulgatum* is used as a reference in the following descriptions. Characteristics of the other species were compared to this reference and differences were recorded.

The following section presents a mixture of description and interpretation. However, stricter separation of these aspects would not support a clear and easily understandable presentation of the results. For establishing our homology hypotheses we supplemented our data with information from Maloeuf [9], Asahina [1], Ninomiya and Yoshizawa [22] and Büsse et al. [4], focusing on Asahina’s [1] comprehensive study of *Epiophlebia superstes*. This species represents a conspicuous mixture of anisopteran and zygopteran characters [1,4,7,64]. Furthermore, in many aspects *Epiophlebia* seems to represent the most ancestral character distribution within the Odonata e.g. [65].

For the skeletal elements of the thorax, the nomenclature of Asahina [1] is used. Where necessary, this is supplemented with terms from Snodgrass [66] and Ninomiya & Yoshizawa [14]. A table listing all muscles, including their attachment points, is available as an additional data file (Additional file 1). A table comparing our results with data from several other publications is available as Additional file 2. Furthermore, an interactive 3D-PDF (Additional file 3) as well as a cross, a sagital and a horizontal section (Additional file 4) assists for a deeper understanding.

For naming the thoracic muscles of Odonata the nomenclature of Friedrich & Beutel [62] and the homologizations of Büsse et al. [4] were used where possible. Otherwise, a new name following the system of Friedrich & Beutel [62] was generated and marked with * in the following descriptions. The numbers in parentheses correspond to Asahina’s [1] nomenclature for Odonata. An additional number in parentheses within the first set denotes the homologous muscle of the meso- or metathorax.

### Musculature of the prothorax

**Figure 1:**
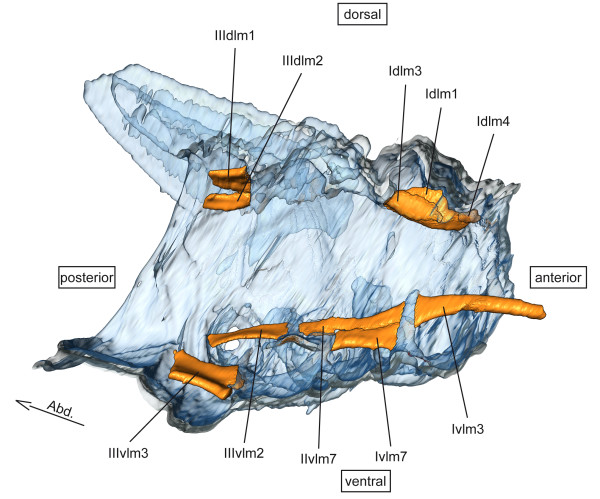
**Dorsal longitudinal and ventral longitudinal musculature of *Sympetrum vulgatum.*** 3D - reconstruction from SRμCT data showing the left half of the thorax. Abd - Abdomen, dlm - dorsal longitudinal muscle, vlm - ventral longitudinal muscle.

**dlm – dorsal longitudinal muscles** (Figure 1)

**Idlm1** – Musculus prophragma-occipitalis (3)

Origin: Apex of tergal apophysis 2.

Insertion: Median at the postocciput.

Characteristics: Runs ventral to Idlm3. The point of origin in *Aeshna affinis* is laterally at the caudal edge of tergite1.

**Idlm3** – M. prophragma-cervicalis (2)

Origin: Tergal apophysis 1.

Insertion: Base of tergal apophysis 2.

Characteristics: Runs dorsal to Idlm1.

**Idlm4** – M. cervico-occipitalis dorsalis (1)

Origin: Tergal apophysis 1.

Insertion: Median at postocciput.

Characteristics: Same direction as Idlm3. Comparatively small and short in *Cordulegaster bidentatus*.

**Figure 2:**
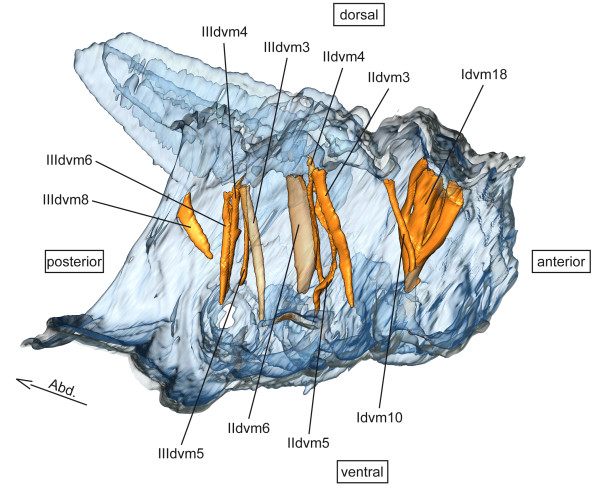
**Dorso-ventral musculature of *Sympetrum vulgatum*.** 3D - reconstruction from SRμCT data showing the left half of the thorax. Abd - Abdomen, dvm - dorso-ventral muscle.

**dvm – dorsoventral muscles** (Figure 2)

**Idvm10** – M. profurca-phragmalis (20)

Origin: Apex of profurca.

Insertion: Apex of tergal apophysis 2.

Characteristics: Intersegmental muscle and homologous to IIIdvm8. Nymphal muscle e.g. [1].

**Idvm15** – M. propleuro-coxalis superior (13)

Origin: Antero-lateral part of tergite 1.

Insertion: Anterior procoxal rim.

Characteristics: Same point of insertion as Itpm9.

**Idvm18** – M. pronto-coxalis lateralis (14 & 15)

Origin: Postero-lateral part of tergite 1.

Insertion: Lateral procoxal disk.

Characteristics: By far the largest muscle in the prothorax.

**Figure 3:**
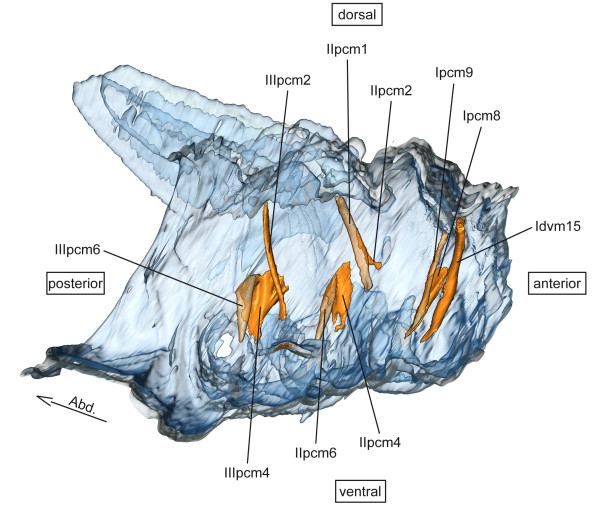
**Pleuro-coxal musculature of *Sympetrum vulgatum*.** 3D - reconstruction from SRμCT data showing the left half of the thorax. Abd - Abdomen, pcm - pleuro-coxal muscle.

**pcm – pleuro-coxal muscles** (Figure 3)

**Ipcm8** – M. propleuro-trochanteralis (18)

Origin: Dorso-median part of Episternum 1.

Insertion: Tendon of protrochanter.

Characteristics: Inserts at the same tendon as Ipcm9 and Iscm6.

Ipcm9* – M. protergro- trochanteralis (17)

Origin: Lateral part of tergite 1, close to the pleura.

Insertion: Tendon of protrochanter.

Characteristics: Inserts at the same tendon as Ipcm8 and Iscm6, homolog to IIpcm5 and IIIpcm5 (see Discussion).

**Figure 4:**
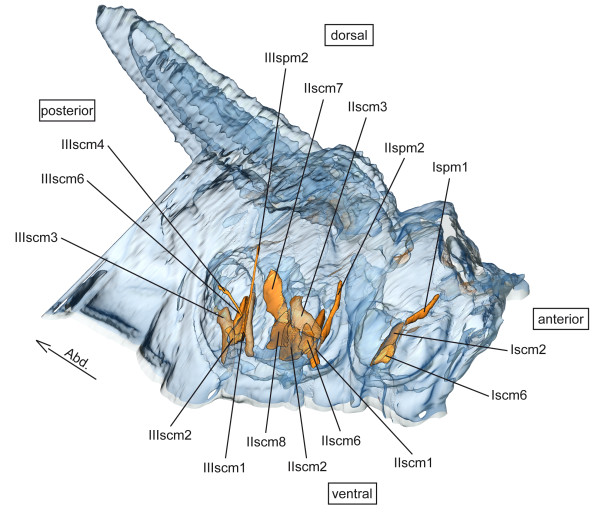
**Sterno-coxal and sterno-pleural musculature of *Sympetrum vulgatum*.** 3D - reconstruction from SRμCT data showing the left half of the thorax. Abd - Abdomen, scm - sterno-coxal muscle, spm - sterno-pleural muscle.

**scm – sterno-coxal muscles** (Figure 4)

**Iscm2** – M. profurca-coxalis posterior (16)

Origin: External side of the base of profurca.

Insertion: Posterior procoxal rim.

Characteristics: Same point of origin as Iscm6.

**Iscm6** – M. profurca-trochanteralis (19)

Origin: External side of the base of profurca.

Insertion: Tendon of protrochanter.

Characteristics: Inserts at the same tendon as Ipcm9 and Ipcm8.

**spm – sterno-pleural muscles** (Figure 4)

**Ispm1** – M. profurca-apodemalis (new for Odonata)

Origin: Apex of profurca.

Insertion: Apodem of propleura.

Characteristics: Homologous to the meso- and metathoracal muscles IIspm2 and IIIspm2.

**Figure 5:**
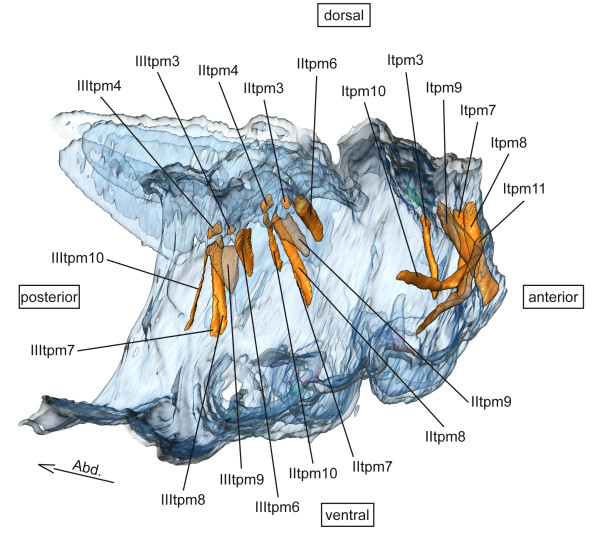
**Tergo-pleural musculature of *Sympetrum vulgatum*.** 3D - reconstruction from SRμCT data showing the left half of the thorax. Abd - Abdomen, tpm - tergo-pleural muscle.

**tpm – tergo-pleural muscles** (Figure 5)

**Itpm3** – M. pronoto-pleuralis anterior (12)

Origin: Lateral side of tergite 1.

Insertion: Anterior part of episternum 1.

Characteristics: Minute muscle.

Itpm7* – M. protergo-cervicalis posterior (4)

Origin: Lateral part of tergite 1.

Insertion: Lateral of cervix membrane.

Characteristics: Runs posterior to Itpm8.

Itpm8* – M. protergo-cervicalis anterior (5)

Origin: Most antero-lateral part of tergite 1.

Insertion: Lateral of cervix membrane.

Characteristics: Runs anterior to Itpm7. Most antero-lateral muscle in the prothorax; in *A. affinis* the point of insertion is very close to the boundary of the caput.

Itpm9* – M. protergo-preepisternalis (7)

Origin: Tergite 1, lateral of tergal apophysis 2.

Insertion: Base of preepisternal apodem 1.

Characteristics: Origin is located postero-lateral of Itpm7 and Itpm8.

Itpm10* – M. prosterna-coxalis dextra (9)

Origin: Apex of right preepisternal apodem 1.

Insertion: Anterior of left procoxal rim.

Characteristics: Intersects with muscle Itpm11, more or less in the middle of the anterior part of the prothorax.

Itpm11* – M. prosterna-coxalis sinister (10)

Origin: Apex of left preepisternal apodem 1.

Insertion: Anterior of right procoxal rim.

Characteristics: Intersects with muscle Itpm10, more or less in the middle of the anterior part of the prothorax.

**vlm – ventral longitudinal muscles** (Figure 1)

**Ivlm3** – M. profurca-tentorialis (11)

Origin: Apex of profurca.

Insertion: Cranial at the tentorial bar.

Characteristics: Runs into the head capsule.

**Ivlm7** – M. profurca-mesofurcalis (41)

Origin: Anterior part of furca-branch 2.

Insertion: Posterior part of furca 1.

Characteristics: In Asahina [1] a muscle of the mesothorax.

### Musculature of the pterothorax

The meso- and metathorax of Odonata are coalesced into a functional unit, the synthorax [67]. The pleurotergal region of the thorax of anisopteran nymphs is less sclerotized it is in the adults. In this area of the thorax the wings develop during ontogenesis [68].

### Musculature of the mesothorax

**dlm – dorsal longitudinal muscles** (Figure 1)

**IIdlm1** (25 (45)) – M. prophragma-mesophragmalis

Origin: Tergal apophysis 3.

Insertion: Tergal aposphysis 4.

Characteristics: Minute muscle.

**dvm – dorsoventral muscles** (Figure 2)

**IIdvm1**- M. mesonoto-sternalis (23′ (46′))

Origin: Base of mesofurca.

Insertion: Postero-lateral edge of mesothoracic wing bud.

Characteristics: Inserts distal of IIdvm3; not recognizable in *S. vulgatum*.

**IIdvm3**- M. mesonoto-trochantinalis posterior (23 (46))

Origin: Base of mesofurca.

Insertion: Antero-lateral edge of mesothoracic wing bud.

Characteristics: Same point of insertion as IIdvm4 and IIdvm5.

**IIdvm4** - M. mesonoto-coxalis anterior (26 (48))

Origin: Anterio-lateral edge of mesocoxa.

Insertion: Antero-lateral edge of mesothoracic wing bud.

Characteristics: Same point of insertion as IIdvm3 and IIdvm5.

**IIdvm5** - M. mesonoto-coxalis posterior (27 (49))

Origin: Lateral at the mesocoxal disk.

Insertion: Antero-lateral edge of mesothoracic wing bud.

Characteristics: Same point of insertion as IIdvm3 and IIdvm4. Single-branched in *S. vulgatum* cf. [1]. In *A. affinis* and *C. bidentatus* it is a dichotomous muscle cf. [9].

**IIdvm6** - M. mesocoxa-subalaris (37 (60))

Origin: Lateral part of tergite 2.

Insertion: Postero-lateral apodem of mesocoxa.

Characteristics: Strongest muscle in the mesothorax of *S. vulgatum*.

**pcm – pleuro-coxal muscles** (Figure 3)

**IIpcm1 -** M. mesanepisterno-trochantinalis (21 (43))

Origin: Preepisternum 2, close to the intersegmental border.

Insertion: Lateral of the tergal apophysis 2 at tergite 2.

Characteristics: Same point of insertion as IIpcm2, it is the strongest muscle in the mesothorax of *A. affinis* and *C. bidentatus*.

**IIpcm2 -** M. mesobasalare-trochantinalis (22 (44))

Origin: Base of preepisternal apodem 2.

Insertion: Lateral of the tergal apophysis 2 at tergite 2.

Characteristics: Same point of insertion as IIpcm1; minute muscle.

**IIpcm4** - M. mesanepisterno-coxalis posterior (36 (58))

Origin: At the base of the interpleural ridge 2.

Insertion: Antero-external part of mesocoxa.

Characteristics: The muscle runs far laterally – connected very close to the pleuron.

**IIpcm6** - M. mesopleura-trochanteralis (39 (62))

Origin: Dorsal part of katepisternum 2.

Insertion: Tendon of mesotrochanter.

Characteristics: Same tendon as IIscm6.

**scm – sterno-coxal muscles** (Figure 4)

**IIscm1** - M. mesofurca-coxalis anterior (new for Odonata)

Origin: Lateral base of mesofurca.

Insertion: Antero-external ridge of mesocoxa.

Characteristics: Postero-lateral of IIpcm4.

**IIscm2** - M. mesofurca-coxalis posterior (new for Odonata)

Origin: Lowermost part of mesofurca.

Insertion: Postero-lateral apodem of mesocoxa.

Characteristics: Point of origin is ventral to IIscm6 and point of insertion ventral to IIscm3.

**IIscm3** - M. mesofurca-coxalis medialis (38 (61))

Origin: Lateral base of mesofurca.

Insertion: Postero-lateral apodem of mesocoxa.

Characteristics: Runs medially to IIdvm6.

**IIscm6** - M. mesofurca-trochanteralis (40 (63))

Origin: Latero-external side of mesofurca.

Insertion: Tendon of mesotrochanter.

Characteristics: Same tendon as IIpcm6.

**IIscm7** - M. mesospina-metacoxalis (59)

Origin: Preepisternal apodem.

Insertion: Antero-lateral edge of metacoxa.

Characteristics: Intersegmental muscle. Asahina [1] described this muscle as a nymphal muscle of the metathorax.

IIscm8* - M. mesospina-mesocoxalis (new for Odonata)

Origin: Medio-ventral part of preepisternal apodem.

Insertion: Postero-lateral at mesocoxa, close to IIdvm6.

Characteristics: Funnel-shaped muscle.

**spm – sterno-pleural muscles** (Figure 4)

**IIspm2** – M. mesofurca-pleuralis (35 (57))

Origin: Apex of mesofurca.

Insert: Interpleural ridge 2.

Characteristics: Nymphal muscle e.g. [1].

**tpm – tergo-pleural muscles** (Figure 5)

**IItpm3** – M. mesonoto-basalaris (new for Odonata)

Origin: Dorsal side of mesothoracic wing bud, anterior to the origin of IItpm4.

Insertion: Ventral side of mesothoracic wing bud, anterior to origin of IItpm4.

Characteristics: Runs within the wing bud, anterior to IItpm4.

**IItpm4** – M. mesonoto-pleuralis anterior (28 (50))

Origin: Dorsal side of mesothoracic wing bud, posterior to the origin of IItpm3.

Insertion: Ventral side of mesothoracic wing bud, posterior to the origin of IItpm3.

Characteristics: Runs within the wing bud, posterior to IItpm3.

**IItpm6** - M. mesonoto-pleuralis posterior (31 (53))

Origin: Upper part of interpleural ridge 2.

Insertion: Antero-dorsal edge of mesothoracic wing bud.

**IItpm7** - M. mesanepisterno-axillaris (33 (55))

Origin: Ventral part of epimeron 2.

Insertion: Lateral edge of mesothoracic wing bud.

Characteristics: Runs between IItpm8 (anterior) and IItpm10 (posterior)

**IItpm8** - M. mesepimero-axillaris secundus (32 (54))

Origin: Ventral part of epimeron 2.

Insertion: Lateral edge of mesothoracic wing bud.

Characteristics: Anterior to IItpm8 and IItpm10.

**IItpm9** – M. mesepimero-axillaris tertius (29/30 (51/52))

Origin: Dorsal part of epimeron 2.

Insertion: Inner side of ventral part of mesothoracic wing bud.

Characteristics: Only one muscle is recognizable (see Discussion)

**IItpm10** – M. mesepimero-subalaris (34 (56))

Origin: At the base of the interpleural ridge 2.

Insertion: Lateral edge of mesothoracic wing bud.

Characteristics: Posterior to IItpm7 and IItpm8.

**vlm – ventral longitudinal muscles** (Figure 1)

**IIvlm6** - M. mesospina-abdominosternalis (68)

Origin: Posterior part of preepisternal apodem 3.

Insertion: Antecostal apodem e.g. [9].

Characteristics: This muscle runs from the mesothorax into the abdomen.

**IIvlm7** - M. mesofurca-abdominosternalis (42 (64))

Origin: Posterior at the mesofurca.

Insertion: Within the abdomen; acoording to Asahina [1] at the anterior margin of first abdominal sternite.

Characteristics: Runs from the mesothorax through the metathorax into the abdomen.

### Muscles of the metathorax

**dlm – dorsal longitudinal muscles** (Figure 1)

**IIIdlm1** - M. mesophragma-metaphragmalis (45(25))

Origin: Tergal apophysis 4.

Insertion: Transversal ridge between abdomen and thorax, sternal part.

Characteristics: Dorso-ventral of IIIdlm2.

**IIIdlm2** - M. metanoto-phragmalis (45′)

Origin: Tergal apophysis 4.

Insertion: Transversal ridge between abdomen and thorax, sternal part.

Characteristics: Ventro-lateral of IIIdvm1.

**dvm – dorsoventral muscles** (Figure 2)

**IIIdvm1**- M. mesonoto-sternalis (46′ (23′))

Origin: Base of metafurca.

Insertion: Postero-lateral edge of metathoracic wing bud.

Characteristics: Inserted distal of IIIdvm3. Not present in *S. vulgatum*.

**IIIdvm3** - M. metanoto-trochantinalis (46 (23))

Origin: Base of metafurca.

Insertion: Antero-lateral edge of metathoracic wing bud.

Characteristics: Same point of insertion as IIIdvm4 and IIIdvm5.

**IIIdvm4** - M. metanoto-coxalis anterior (48 (26))

Origin: Anterio-lateral edge of metaocoxa.

Insertion: Antero-lateral edge of metathoracic wing bud.

Characteristics: Same point of insertion as IIIdvm3 and IIIdvm5.

**IIIdvm5** - M. metanoto-coxalis posterior (49 (27))

Origin: Median part of metacoxal disk.

Insertion: Antero-lateral edge of metathoracic wing bud.

Characteristics: Same point of insertion as IIIdvm3 and IIIdvm4. It is a single-branched muscle, not dichotomous like the homologous mesothoracic muscle IIdvm5 e.g. [1,9].

**IIdvm6** - M. metacoxa-subalaris (60(37))

Origin: Lateral part of tergite 3.

Insertion: Postero-lateral part of metacoxa.

Characteristics: Strongest muscle in the metathorax of *S. vulgatum*.

**IIIdvm8** - M. metanoto-phragmalis (67)

Origin: Dorsal part of the posterior ridge of epimeron 3.

Insertion: Posterior end of metafurca.

**pcm – pleuro-coxal muscles** (Figure 3)

**IIIpcm1 -** M. metanepisterno-trochantinalis (43 (21))

Origin: Preepisternum 3.

Insertion: Lateral of the tergal apophysis 3 at tergite 3.

Characteristics: Same point of insertion as IIIpcm2. This is the strongest muscle in the metathorax of *A. affinis* and *C. bidentatus*.

**IIIpcm2 -** M. metabasalare-trochantinalis (44(22))

Origin: Base of preepisternal apodem 3.

Insertion: Lateral of the tergal apophysis 3 at tergite 3.

Characteristics: Minute muscle with the same point of insertion as IIpcm1.

**IIIpcm4** - M. metanepisterno-coxalis posterior (58 (36))

Origin: Dorsal part of interpleuralridge 3.

Insertion: Antero-external part of metacoxa.

Characteristics: The muscle runs laterally.

**IIIpcm6** - M. mesopleura-trochanteralis (39 (62))

Origin: Dorsal part of katepisternum 3.

Insertion: Tendon of metatrochanter.

Characteristics: Same tendon as IIIscm6. In *A. affinis* and *C. bidentatus* the origin nearly covers the whole katepisternum.

**scm – sterno-coxal muscles** (Figure 4)

**IIIscm1** - M. metafurca-coxalis anterior (new for Odonata)

Origin: Lateral base of metafurca.

Insertion: Antero-external ridge of metacoxa.

Charecteristics: Postero-lateral of IIIpcm4.

**IIIscm2** - M. metafurca-coxalis posterior (new for Odonata)

Origin: Lowermost part of metafurca.

Insertion: Postero-lateral apodem of metacoxa.

Charecteristics: Point of origin is ventral to IIIscm6 and point of insertion ventral to IIIscm3.

**IIIscm3** - M. metafurca-coxalis medialis (61(38))

Origin: Lateral base of metafurca.

Insertion: Postero-lateral apodem of metacoxa.

Characteristics: Runs medially to IIIdvm6.

**IIIscm4** - M. metafurca-coxalis lateralis (new for Odonata)

Origin: Apex of metafurca.

Insertion: Lateral base of metacoxa, at the border of pleurite

Characteristics: Very thin and far lateral running muscle.

**IIIscm6** - M. metafurca-trochanteralis (63 (40))

Origin: Latero-external side of metafurca.

Insertion: Tendon of metatrochanter.

Characteristics: Same tendon as IIIpcm6.

**spm – sterno-pleural muscles** (Figure 4)

**IIIspm2** – M. metafurca-pleuralis (57 (35))

Origin: Apex of metafurca.

Insert: Median part of interpleural ridge 3.

Characteristics: Nymphal muscle e.g. [1].

**tpm – tergo-pleural muscles** (Figure 5)

**IIItpm3** – M. metanoto-basalaris (new for Odonata)

Origin: Dorsal side of metathoracic wing bud, anterior to the origin of IIItpm4.

Insertion: Ventral side of metathoracic wing bud, anterior to origin of IIItpm4.

Characteristics: Runs within the wing bud, anterior to IIItpm4.

**IIItpm4** – M. metanoto-pleuralis anterior (50 (28))

Origin: Dorsal side of metathoracic wing bud, posterior to the origin of IIItpm3.

Insertion: Ventral side of metathoracic wing bud, posterior to the origin of IIItpm3.

Characteristics: Runs within the wing bud, posterior to IIItpm3

**IIItpm6** - M. metanoto-pleuralis posterior (53 (31))

Origin: Upper part of interpleural ridge 3.

Insertion: Antero-dorsal edge of metathoracic wing bud.

**IIItpm7** - M. metanepisterno-axillaris (55 (33))

Origin: Ventral part of epimeron 3.

Insertion: Lateral edge of metathoracic wing bud.

Characteristics: Runs between IIItpm8 (anterior) and IIItpm10 (posterior).

**IIItpm8** - M. metapimero-axillaris secundus (54 (32))

Origin: Ventral part of epimeron 3.

Insertion: Lateral edge of metathoracic wing bud.

Characteristics: Anterior to IIItpm8 and IIItpm10.

**IIItpm9** – M. metapimero-axillaris tertius (51/52 (29/30))

Origin: Dorsal part of epimeron 3.

Insertion: Inner side of ventral part of metathoracic wing bud.

Characteristics: Only one muscle is recognizable (see discussion).

**IIItpm10** – M. metapimero-subalaris (56 (34))

Origin: Median part of interpleural ridge 3.

Insertion: Lateral edge of metathoracic wing bud.

Characteristics: Posterior to IIItpm7 and IIItpm8.

**vlm – ventral longitudinal muscles** (Figure 1)

**IIIvlm2** - M. mesofurca-abdominosternalis (65)

Origin: Posterior part of metafurca, close to the prefurca invagination.

Insertion: Within the abdomen, second abdominal sternite.

Characteristics: Not identifiable in *S. vulgatum*.

**IIIvlm3** - M. metaspina-abdominosternalis (66)

Origin: Posterior part of sternum 3.

Insertion: Within the abdomen, second abdominal sternite.

Characteristics: This muscle is caudal distinctly flattened.

## Discussion

### Musculature of the prothorax

**dlm – dorsal longitudinal muscles** (Figure 1)

The tergal apophyses of Odonata are not homologous to the primary phragmata [69], but rather to the pseudo phragmata [67]. Therefore, the homology of Idlm1, Idlm3 and Idlm4 is unquestionable cf. [62].

**dvm – dorsoventral muscles** (Figure 2)

The muscle Idvm10 is only present in odonatan nymphs [1,9]. Because of the correspondence of the pseudo phragmata with the tergal apophysis, assuming a homology with the neopteran muscle is straightforward. Due to identical attachment points cf. [62] the same is true for the homology of Idvm15 and Idvm18. Maloeuf [9] and Asahina [1] described Idvm18 as two muscles: M14 and an independent nymphal muscle M15. At least in Anisoptera it is more likely that this is just one muscle split into several bundles. This muscle is very strong in nymphs, which probably is related to their walking lifestyle and the pleural part of this coxal remoter [9] seems to be unique in nymphs.

**pcm – pleuro-coxal muscles** (Figure 3)

The points of origin of Ipcm8 and Ipcm9, at the episternum 1 and at the lateral part of tergite 1, are slightly relocated in comparison to where Asahina [1] described them for *Epiophlebia* at the epimeron 1 and at the median lobe of tergite 1, respectively. However, the neopteran homologue of Ipcm8 has similar attachment points cf. [62]. Muscle Ipcm9 has no homologous muscle in the generalized Neoptera thorax [62]. It seems to represents a unique odonatan muscle. The muscle Ipcm9 was interpreted as pleural muscles, even though its origin is on the tergite, because of the positions of its homologous muscles IIpcm5 and IIIpcm5 in the neopteran mesothorax and metathorax cf. [62]. This seems to be an interesting evolutionary trade-off, because IIpcm5 and IIIpcm5 are not present in Odonata. The pterygote ground pattern might show Ipcm9, IIpcm5 and IIIpcm5 as homologous muscles in pro-, meso- and metathorax. During the evolution of Odonata IIpcm5 and IIIpcm5 were reduced, whereas Ipcm9 was reduced during the evolution of the Neoptera.

**scm – sterno-coxal muscles** (Figure 4)

The attachment points of Iscm2 and Iscm6 are congruent with *Epiophlebia*[1] as well as with their neopteran counterparts cf. [62].

**spm – sterno-pleural muscles** (Figure 4)

The muscle Ispm1 is new for Odonata. Neither Asahina [1] nor Maloeuf [9] described it. The attachment points coincide with those of its neopteran homologue cf. [62].

**tpm – tergo-pleural muscles** (Figure 5)

Itpm3 has been homologized with its neopteran counterpart, because it has the same attachment points cf. [62]. The muscles Itpm7, Itpm8, Itpm9, Itpm10 and Itpm11 have no counterparts in the thorax of Neoptera. Some of the attachment points of the muscles Itpm7- Itpm11 differ slightly from the descriptions of Maloeuf [9] and Asahina [1].

**vlm – ventral longitudinal muscles** (Figure 1)

Asahina [1] described Ivlm7 under the name M41 as a muscle of the mesothorax, whereas Maloeuf [9] named it M42 in the prothorax.

### Musculature of the pterothorax

**dlm – dorsal longitudinal muscles** (Figure 1)

As in the prothorax, the homology of IIdlm1 (IIIdlm1) is unequivocal cf. [62], because of the position of its attachment points on the tergal apophyses. Even though IIIdlm2 has no homologue in the mesothorax, its attachment points are congruent with those of its counterpart in the Neoptera, which supports our homology hypothesis. According to Büsse et al. [4] muscle IIIdlm2 is present only in Zygoptera and *Epiophlebia*; this assumption can be refuted, at least for the nymphs.

**dvm – dorsoventral muscles** (Figure 2)

The attachment points of IIdvm4 (IIIdvm4) and IIdvm5 (IIIdvm5) as well as the points of insertion of IIdvm1 (IIIdvm1) and IIdvm3 (IIIdvm3) are identical [62], because the wing buds are a subset of the notum and are inverted during metamorphosis. Thus, areas that are oriented ventrally in the nymphs represent the tergal structures especially the wing base sclerites, and the dorsal, or external, part of the wing bud represent the most dorsal area of the pleuron in the adult. The points of origin and insertion of the adult zygopteran dorso-ventral muscles are usually shifted to some degree in comparison to Neoptera [4], because of the changes in shape and size of the notum in Odonata. The points of origin of IIdvm1 (IIIdvm1), IIdvm3 (IIIdvm3) and IIdvm6 (IIIdvm6) differ slightly from Neoptera cf. [62] but these relocations do not affect the function of these muscles as elevators of the wings [4].

Muscle IIdvm1 (IIIdvm1) is not identifiable in *S. vulgatum*, but distinct in *A. affinis* and *C. bidentatus*. Muscle IIdvm5 is single-branched in *S. vulgatum* (as described in [1]) and dichotomous in *A. affinis* and *C. bidentatus* cf. [9]. Muscle IIIdvm5 is single-branched in all species studied.

Muscle IIIdvm8 has no homologue in the mesothorax; the insertion is identical to the Neoptera cf. [62]. The point of origin at the edge of epimeron 3 corresponds to the neopteran metaphragma [66].

**pcm – pleuro-coxal muscles** (Figure 3)

The points of origin of IIpcm4 (IIIpcm4) and IIpcm6 (IIIpcm6) at the pleuron differ slightly. In muscles IIpcm1 and IIpcm2 (IIIpcm2) the functional attachment point (point of insertion) is slightly relocated dorsally from the basalare, to the anepisternum and to the tergalapophysis 2, respectively, due to their function as direct flight muscles [4,9]. These structures to which the muscles are attached in Odonata and other Pterygota are not homologous, but the function as flight muscles is retained and due to the parsimony principle a homology is likely.

Muscle IIpcm1 (IIIpcm1) is the strongest muscle in the mesothorax of *A. affinis* and *C. bidentatus*, whereas in *S. vulgatum* it is IIdvm6. Furthermore, muscle IIpcm1 (IIIpcm1) was described as M21 (M43) by Asahina [1] and as M22 (M44) by Maloeuf [9]. Muscle IIvpcm2 (IIIpcm2) is M22 (M44) in Asahina [1] and M21 (M43) in Maloeuf [9].

**scm – sterno-coxal muscles** (Figure 4)

The muscles IIscm1 (IIIscm1), IIscm2 (IIIscm2) and IIscm8 are new for Odonata; neither Maloeuf [9], Asahina [1] nor Büsse et al. [4] mentioned them. The muscles IIscm1 (IIIscm1) and IIscm2 (IIIscm2) are not present in adult Zygoptera [4]. Therefore, they could represent either unique muscles of Odonata nymphs or unique muscles of Anisoptera (or Epiprocta); even a combination is conceivable. However, the homologies of IIscm1, IIscm2, IIscm3, IIscm6 and IIscm7 are well supported by their positions in relation to other structures in the thorax. Muscle IIscm8 is not present in the generalized neopteran thorax [cf. 62]. Furthermore, this muscle has no counterpart in the pro- or metathorax. Asahina [1] described muscle IIscm7 as a nymphal muscle. Our results and its absence in adult Zygoptera [4] confirm this interpretation. Muscle IIscm7 has no homologous muscle in the metathorax. Muscle IIIscm4 has no homologue in the mesothorax.

**spm – sterno-pleural muscles** (Figure 4)

Asahina’s [1] interpretation of muscle IIspm2 (IIIspm2) as a nymphal muscle is supported by our results and its absence from adult Zygoptera [4].

**tpm – tergo-pleural muscles** (Figure 5)

Muscle IItpm3 (IIItpm3) is new for Odonata. All tergo-pleural muscles have at least one attachment point within the wing bud. The homologizations are quite straightforward, because of the inversion of the wing buds during metamorphosis (see also dvm above). The points of origin of muscles IItpm6 (IIItpm6) – IItpm10 (IIItpm10) are slightly relocated. For example muscle IItpm6 originates at the interpleural ridge in Odonata and at the pleural arm in Neoptera cf. [62]. However, the orientation and especially the function of these muscles are preserved. Muscle IItpm9 (IIItpm9) was described as two muscles lying very close to each other [1,4,9]; this could not be confirmed. Muscle IItpm2 (IIItpm2), which is present in adult Zygoptera [4], could not be confirmed. It might be a unique muscle for Zygoptera [cf. 9, 1].

**vlm – ventral longitudinal muscles** (Figure 1)

Muscle IIvlm7 is one of the most conspicuous muscles. It originates in the mesothorax, runs through the metathorax and inserts in the abdomen. Due to this characteristic course and the identical attachment points, the homologization is straightforward cf. [62]. The homologies of IIIvlm2 and IIIvlm3 are equally unequivocal; both have no homologue in the mesothorax. Asahina’s [1] description of IIvlm6 is unclear; however, the figures of Maloeuf [9] and Asahina [1] are conclusive. Muscle IIvlm6 belongs to the mesothorax not to the metathorax as it had been described cf. [1,9]. Muscle Ivlm7 was described as M41 by Asahina [1] and as M42 by Maloeuf [9]. IIvlm7 is M42 in Asahina [1] and M41 in Maloeuf [9].

## Conclusions

The homologization of the thoracic musculature of Odonata with the generalized neopteran thorax and the established nomenclature of Friedrich & Beutel [62] was surprisingly straightforward [4]. The simplicity of our hypothesis is distinctly positive. In accordance with the “parsimony principle” or “Ockham’s razor” e.g. [70-72], we tried to find the hypothesis that requires the smallest amount of assumptions to explain the observations. Since we assume that Pterygota are monophyletic (which is supported by numerous phylogenetic analyses), we also have to assume that there once existed a last common ancestor of Pterygota that also represents its morphological ground pattern. From this ground pattern the evolution of all pterygote subgroups started and therefore there also should be a pattern of homologies between these subgroups. Starting from a ground pattern of Dicondylia (wingless insects), a thoracic musculature comprising a high number of muscles is most likely. Zygentoma and Archaeognatha show an excessive number of muscles with just a few stands each in their thorax e.g. [73-75]. Our findings support the assumption of a high number of muscles in the thorax of the last common ancestor of Pterygota. We found six muscles that are not known for Neoptera. It seems quite probable that these muscles (Ipcm9, Itpm7 – Itpm11) were retained from the pterygote ground pattern. For example, muscle Ipcm9 has homologous muscles in the neopteran pterothorax (IIpcm5 and IIIpcm5; Figure 3) cf. [62]. This seems to be an interesting evolutionary trade-off, since IIpcm5 and IIIpcm5 are not present in Odonata. The pterygote ground pattern might show Ipcm9, IIpcm5 and IIIpcm5 as homologous muscles in pro-, meso- and metathorax. During the evolution of Odonata IIpcm5 and IIIpcm5 were reduced and, on the other hand, Ipcm9 was reduced during the evolution of the Neoptera.

The present study explicitly focuses on the question of the homology and evolution of the thoracic muscles of Pterygota. Establishing muscle homologies among the pterygote taxa is essential for subsequent application of musculature characters in phylogenetic analyses of Pterygota. The results and interpretations presented herein represent a significant advancement in this area of study.

## Methods

The late instar nymphs of the Anisoptera species *Sympetrum vulgatum* (Linnaeus, 1758) (Libellulidae), *Aeshna affinis* Van Der Linden, 1820 (Aeshnidae) and *Cordulegaster bidentatus* Sélys, 1843 (Cordulegasteridae) used for this investigation where taken from the collection of the Johann-Friedrich-Blumenbach-Institute of Zoology & Anthropology of the Georg-August-University in Göttingen, Germany. All regulations concerning the protection of free-living species have been followed.

The specimens were fixed in an alcoholic Bouin solution (= Duboscq-Brasil) [76] and subsequently stored in 70% ethanol.

For the investigation of *Sympetrum vulgatum*, synchrotron radiation micro computed tomography (SRμCT) was applied in order to generate data for the three-dimensional reconstruction of the structures of interest. Function and construction of a synchrotron have been described by Betz et al. [77]. The data were generated at the Deutsches Elektronen Synchrotron (DESY) in Hamburg (Germany), using the beamline BW2, (Proposal no. I-20090102, Aug. 2009, SB) and at the Swiss Light Source (SLS) in Villingen (Switzerland), using the beamline TOMCAT, (Proposals no. 20080794, Mai 2009 and no. 20100088, Nov. 2010, TH).

Processing and visualization of the data were done with VGS Amira® 5.2. (Visage Imaging, Richmond, Australia).

For freehand preparation the specimens were halved along the body axis with a razorblade. The right side of the body was pasted into Paraplast, to preserve the shapes of the body during further preparations. Subsequently, the gut and all of the other tissues except the musculature were removed. For investigation and drawing, a Zeiss Stemi SV11 stereomicroscope with an attached camera lucida was used.

All figures were subsequently processed in Photoshop CS3, version 10.0.1 (Adobe System Inc., San José, USA).

## Competing interest

The authors declared that they have no competing interest.

## Authors’ contributions

SB carried out the morphological studies and generates the 3D reconstruction. SB and TH designed the study and wrote the manuscript. Both authors read and approved the final manuscript.

## Supplementary Material

Additional file 1 **Attachmentpoints of the thorax musculature of *Sympetrum vulgatum.***
Click here for file

Additional file 2 Homologisation of thoracic muscle nomenclatures used by several authors. Click here for file

Additional file 3 **Thorax of *Sympetrum vulgatum.*** A. Cross section of the pterothorax B. Sagital section C. Horizontal section (dorsal view). Cd - coxal disc, dvm - dorso-vetral musculature, Gt - gut, pcm - pleuro-coxal musculature, Pl - pleura, scm - sterno-coxal musculature, St - sternum, TAp - tergal apophysis, tpm – tergopleural musculature, Tr - trachee, vlm – ventral longitudinal musculature, WB - wing buds. Click here for file

Additional file 4 **Model of thoracic musculature of *Sympetrum vulgatum *nymph; reconstructed from SRμCT data showing transparent cuticle and muscles grouped as dorsal longitudinal (dlm), ventral longitudinal (vlm), dorso-ventral (dvm), tergopleural (tpm), sterno-pleural (spm), sterno-coxal (scm) and pleuro-coxal (pcm) muscles.** After clicking the figure the model can be manipulated. Click here for file
