# Supplementary material for: The thorax musculature of Anisoptera (Insecta: Odonata) nymphs and its evolutionary relevance
Source: BMC Evol Biol. 2013 Nov 1;13:237. doi: 10.1186/1471-2148-13-237 (PMC4228402; doi:10.1186/1471-2148-13-237)
Supplement: Additional file 1 — Attachmentpoints of the thorax musculature of Sympetrum vulgatum. [file 1471-2148-13-237-S1.pdf]

Adittional file 1: Attachmentpoints of the thorax musculature of *Sympetrum vulgatum*

Abbrevation	Name	Origin	Insertion
**Prothorax**			
Dorsal longitudinal muscles			
**Idlm1**	Musculus prophragma-occipitalis	Apex of tergal apophysis 2	Median at the postocciput
**Idlm3**	M. prophragma-cervicalis	Tergal apophysis 1	Base of tergal apophysis 2
**Idlm4**	M. cervico-occipitalis dorsalis	Tergal apophysis 1	Median at postocciput
Dorsoventral muscles			
**Idvm10**	M. profurca-phragmalis	Apex of profurca	Apex of tergal apophysis 2
**Idvm15**	M. propleuro-coxalis superior	Anterolateral portion of tergite 1	Anterior procoxal rim
**Idvm18**	M. pronto-coxalis lateralis	Postero-lateral portion of tergite 1	Procoxal disk
Pleuro-coxal muscles			
**Ipcm8**	M. propleuro-trochanteralis	Episternum 1	Tendon of protrochanter
**Ipcm9**	M. protergro-trochanteralis	Lateral portion of tergite 1, close to the pleura.	Tendon of protrochanter
Sterno-coxal muscles			
**Iscm2**	M. profurca-coxalis posterior	External side of the base of profurca	Posterior procoxal rim
**Iscm6**	M. profurca-trochanteralis	External side of the base of profurca	Tendon of protrochanter
Sterno-pleural muscles			
**Ispm1**	M. profurca-apodemalis	Apex of profurca	Apodem of propleura
Tergo-pleural muscles			

**Itpm3**	M. pronoto-pleuralis anterior	Lateral side of tergite 1	Episternum 1
**Itpm7**	M. protergo-cervicalis posterior	Lateral part of tergite 1	Lateral of cervix membrane
**Itpm8**	M. protergo-cervicalis anterior	Most antero-lateral part of tergite 1	Lateral of cervix membrane
**Itpm9**	M. protergo-preepisternalis	Tergite 1, lateral of tergal apophysis 2	Base of preepisternalapodem 1
**Itpm10**	M. prosterna-coxalis dextra	Apex of right preepisternalapodem 1	Anterior of left procoxal rim
**Itpm11**	M. prosterna-coxalis sinister	Apex of left preepisternalapodem 1	Anterior of right procoxal rim
Ventral longitudinal muscles			
**Ivlm3**	M. profurca-tentorialis	Apex of profurca	Cranial tentorial bar
**Ivlm7**	M. profurca-mesofurcalis	Furca-branch 2	Furca 1
**Mesothorax**			
Dorsal longitudinal muscles			
**IIdlm1**	M. prophragma-mesophragmalis	Tergal apophysis 3	Tergal aposphysis 4
Dorsoventral muscles			
**IIdvm1**	M. mesonoto-sternalis	Base of Mesofurca	Postero-lateral edge of mesowing bud
**IIdvm3**	M. mesonoto-trochantinalis posterior	Base of Mesofurca	Antero-lateral edge of mesowing bud
**IIdvm4**	M. mesonoto-coxalis anterior	Anterio-lateral edge of mesocoxa	Antero-lateral edge of mesowing bud
**IIdvm5**	M. mesonoto-coxalis posterior	Mesocoxaldisk	Antero-lateral edge of mesowing bud

**IIdvm6**	M. mesocoxa-subalaris	Lateral part of tergite 2	Postero-lateral apodem of mesocoxa
Pleuro-coxal muscles			
**IIpcm1**	M. mesanepisterno-trochantinalis	Preepisternum 2	Lateral of the tergal apophysis 2 at tergite 2
**IIpcm2**	M. mesobasalare-trochantinalis	Base of preepisternal apodem 2	Lateral of the tergal apophysis 2 at tergite 2
**IIpcm4**	M. mesanepisterno-coxalis posterior	Interpleuralridge 2	Antero-external part of mesocoxa
**IIpcm6**	M. mesopleura-trochanteralis	Dorsal part of Katepisternum 2	Tendon of mesotrochanter
Sterno-coxal muscles			
**IIscm1**	M. mesofurca-coxalis anterior	Lateral base of Mesofurca	Antero-external ridge of mesocoxa
**IIscm2**	M. mesofurca-coxalis posterior	Lowermost part of mesofurca	Postero-lateral apodem of mesocoxa
**IIscm3**	M. mesofurca-coxalis medialis	Lateral base of mesofurca	Postero-lateral apodem of mesocoxa
**IIscm6**	M. mesofurca-trochanteralis	Latero-external side of mesofurca	Tendon of mesotrochanter
**IIscm7**	M. mesospina-metacoxalis	Preepisternal apodem	Anterolateral edge of metacoxa
Sterno-pleural muscles			
**IIspm2**	M. mesofurca-pleuralis	Apex of mesofurca	Interpleural ridge 2
Tergo-pleural muscles			
**IItpm3**	M. mesonoto-basalaris	Dorsal side of mesowing bud, anterior to the origin of IItpm4	Ventral side of mesowing bud, anterior to origin of IItpm4
**IItpm4**	M. mesonoto-pleuralis anterior	Dorsal side of mesowing bud, posterior to the origin of IItpm3	Ventral side of mesowing bud, posterior to the origin of IItpm3

**IItpm6**	M. mesonoto-pleuralis posterior	Upper portion of interpleural ridge 2	Antero-dorsal edge of mesowing bud
**IItpm7**	M. mesanepisterno-axillaris	Ventral part of epimeron 2	Lateral edge of mesowing bud
**IItpm8**	M. mesepimero-axillaris secundus	Ventral part of epimeron 2	Lateral edge of mesowing bud
**IItpm9**	M. mesepimero-axillaris tertius	Dorsal part of epimeron 2	Inner side of ventral portion of mesowing bud
**IItpm10**	M. mesepimero-subalaris	Interpleural ridge 2	Lateral edge of mesowing bud
Ventral longitudinal muscles			
**IIvlm6**	M. mesospina-abdominosternalis	Posterior part of preepisternalapodem 3	Antecostal apodem
**IIvlm7**	M. mesofurca-abdominosternalis	Mesofurca	Within the Abdomen
**Metathorax**			
Dorsal longitudinal muscles			
**IIIdlm1**	M. mesophragma-metaphragmalis	Tergal apophys 4	Transversal ridge between abdomen and thorax
**IIIdlm2**	M. metanoto-phragmalis	Tergal apophysis 4	Transversal ridge between abdomen and thorax
Dorsoventral muscles			
**IIIdvm1**	M. mesonoto-sternalis	Base of Metafurca	Postero-lateral edge of metawing bud
**IIIdvm3**	M. metanoto-trochantinalis	Base of Metafurca	Antero-lateral edge of metawing bud
**IIIdvm4**	M. metanoto-coxalis anterior	Anterio-lateral edge of metaocoxa	Antero-lateral edge of metawing bud
**IIIdvm5**	M. metanoto-coxalis posterior	Metacoxaldisk	Antero-lateral edge of metawing bud

**IIdvm6**	M. metacoxa-subalaris	Lateral part of tergite 3	Postero-lateral part of metacoxa
**IIIdvm8**	M. metanoto-phragmalis	Dorsal portion of the posterior ridge of epimeron 3	Posterior end of metafurca
Pleuro-coxal muscles			
**IIIpcm1**	M. metanepisterno-trochantinalis	Preepisternum 3	Lateral of the tergal apophysis 3 at tergite 3
**IIIpcm2**	M. metabasalare-trochantinalis	Base of Preepisternal apodem 3	Lateral of the tergal apophysis 3 at tergite 3
**IIIpcm4**	M. metanepisterno-coxalis posterior	Interpleuralridge 3	Antero-external part of metacoxa
**IIIpcm6**	M. mesopleura-trochanteralis	Dorsal part of Katepisternum 3	Tendon of metatrochanter
Sterno-coxal muscles			
**IIIscm1**	M. metafurca-coxalis anterior	Lateral base of Metafurca	Antero-external ridge of metacoxa
**IIIscm2**	M. metafurca-coxalis posterior	Lowermost part of metafurca	Postero-lateral apodem of metacoxa
**IIIscm3**	M. metafurca-coxalis medialis	Lateral base of metafurca	Postero-lateral apodem of metacoxa
**IIIscm4**	M. metafurca-coxalis lateralis	Apex of metafurca	Lateral base of metacoxa, at the border of pleurite
**IIIscm6**	M. metafurca-trochanteralis	Latero-external side of metafurca	Tendon of metatrochanter
Sterno-pleural muscles			
**IIIspm2**	M. metafurca-pleuralis	Apex of metafurca	Interpleural ridge 3
Tergo-pleural muscles			
**IIItpm3**	M. metanoto-basalaris	Dorsal side of metawing bud, anterior to the origin of IIItpm4	Ventral side of metawing bud, anterior to origin of IIItpm4

**IIItpm4**	M. metanoto-pleuralis anterior	side of metawing bud, posterior to the origin of IIItpm3	Ventral side of metawing bud, posterior to the origin of IIItpm3
**IIItpm6**	M. metanoto-pleuralis posterior	Upper portion of interpleural ridge 3	Antero-dorsal edgel of metawing bud
**IIItpm7**	M. metanepisterno-axillaris	Ventral part of epimeron 3	Lateral edge of metawing bud
**IIItpm8**	M. metapimero-axillaris secundus	Ventral part of epimeron 3	Lateral edge of metawing bud
**IIItpm9**	M. metapimero-axillaris tertius	Dorsal part of epimeron 3	Inner side of ventral portion of metawing bud
**IIItpm10**	M. metapimero-subalaris	Interpleural ridge 3	Lateral edge of metawing bud
Ventral longitudinal muscles			
**IIIvlm2**	M. mesofurca-abdominosternalis	part of Metafurca (close to the prefurca invagination)	Within the abdomen (second abdominal sternite)
**IIIvlm3**	M. metaspina-abdominosternalis	Poststernum 3	the abdomen (second abdominal sternite)
