# Supplementary material for: The thorax musculature of Anisoptera (Insecta: Odonata) nymphs and its evolutionary relevance
Source: BMC Evol Biol. 2013 Nov 1;13:237. doi: 10.1186/1471-2148-13-237 (PMC4228402; doi:10.1186/1471-2148-13-237)
Supplement: Additional file 2 — Homologisation of thoracic muscle nomenclatures used by several authors. [file 1471-2148-13-237-S2.pdf]

Additional file 2: Homologisation of thoracic muscle nomenclatures used by several authors

"-" absent / "?" uncertain or no information

Friedrich & Beutel (2008)	this study	Büsse et al. (2013)	Asahina (1954)	Willkommen (2008)	Wittig (1955)	Matsuda (1970)
Prothorax						
Idlm1	x	?	3	?	I dlm 10	op-t 3
Idlm2	-	?	-	?	0 dlm 1	op-t 2
Idlm3	x	?	2	?	I dlm 11b	cv(d)-t 1, t 14
Idlm4	x	?	1	?	0 dlm 2	op-t 1
Idlm5	-	?	-	?	I dlm 12	t 12
Idlm6	-	?	-	?	I dlm 12?	t 13
Idvm1	-	?	-	?	0 lm 7	op-cv 1
Idvm2	-	?	-	?	0 lm 7	op-cv 2
Idvm3	-	?	-	?	0 lm 8	op-cv 3
Idvm4	-	?	-	?	0 lm 5	t-s(cv) 1?
Idvm5	-	?	-	?	-	t-cv 1
Idvm6	-	?	-	?	0 lm 6	t-cv 2
Idvm7	-	?	-	?	-	t-cv 3
Idvm8	-	?	-	?	-	t-s(cv) 9
Idvm9	-	?	-	?	-	op-s 2, p-s 3
Idvm10	x	?	-	?	I ism 22	t-s 1
Idvm11	-	?	-	?	I ism 24	t-s 8
Idvm12	-	?	-	?	-	t-s 2
Idvm13	-	?	-	?	I dvm 15	t-ti(cx) 2
Idvm14	-	?	-	?	I dvm 16	t-ti(cx) 3
Idvm15	x	?	13	?	I dvm 17?	t-ti(cx) 1
Idvm16	-	?	-	?	I dvm 19	t-cx 5
Idvm17	-	?	-	?	I dvm 20	t-cx 6, t-cx 7
Idvm18	x	?	14 & 15	?	I dvm 21	t-cx 8
Idvm19	-	?	-	?	I dvm 18	t-tr 1

Additional file 2: Homologisation of thoracic muscle nomenclatures used by several authors

"-" absent / "?" uncertain or no information

Itpm1	-	?	-	?	-	op-p 2
Itpm2	-	?	-	?	0 lm 9	op-p 1, t-p 3
Itpm3	x	?	12	?	I tpm 25	-
Itpm4	-	?	-	?	I tpm 26	t-p 14?
Itpm5	-	?	-	?	I tpm 27?	t-p 15?
Itpm6	-	?	-	?	-	t-p 1, t-p 2
-	Itpm7	?	4	?	?	?
-	Itpm8	?	5	?	?	?
-	Itpm9	?	7	?	?	?
-	Itpm10	?	9	?	?	?
-	Itpm11	?	10	?	?	?
Ispm1	x	?	-	?	I zm 34	p-s1
Ispm2	-	?	-	?	-	p-s 2
Ispm3	-	?	-	?	-	p-s 6
Ispm4	-	?	-	?	-	p-s 7
Ispm5	-	?	-	?	-	p-s 5
Ispm6	-	?	-	?	-	p-s 4
Ispm7	-	?	-	?	-	p-s 10
Ipcm1	-	?	-	?	-	cv-cx 3
Ipcm2	-	?	-	?	-	cv-cx 1, cv-cx 2
Ipcm3	-	?	-	?	-	p-ti(cx) 1
Ipcm4	-	?	-	?	-	p-cx 4
Ipcm5	-	?	-	?	I cpm 28	p-cx 5
Ipcm6	-	?	-	?	-	p-cx 6, p-cx 9
Ipcm7	-	?	-	?	-	p-cx 7
Ipcm8	x	?	18	?	I cpm 29	p-tr 1, p-tr 2
-	Ipcm9	?	17	?	?	?
Ivlm1	-	?	-	?	0 vlm 4	cv-s 1, cv-s 4?
Ivlm2	-	?	-	?	-	op-cv(v) 4

Additional file 2: Homologisation of thoracic muscle nomenclatures used by several authors

"-" absent / "?" uncertain or no information

Ivlm3	x	?	11	?	0 vlm 3	s 1, s 2
Ivlm4	-	?	-	?	I vlm 14	s 14, s16
Ivlm5	-	?	-	?	-	s 17
Ivlm6	-	?	-	?	-	s 15
Ivlm7	x	?	41	?	I vlm 13	s 13
Ivlm8	-	?	-	?	-	s 11
Ivlm9	-	?	-	?	-	s 12
Iscm1	-	?	-	?	I bm 30	s-cx 5
Iscm2	x	?	16	?	I bm 33	s-cx 3
Iscm3	-	?	-	?	I bm 32	s-cx 6
Iscm4	-	?	-	?	-	s-cx 2
Iscm5	-	?	-	?	-	s-cx 4
Iscm6	x	?	19	?	I bm 31	s-tr 1
Iscm7	-	?	-	?	-	s-cx 1, s-cx 7
Mesothorax						
IIdlm1	x	x	25	MT.m	II dlm 35	t 14
IIdlm2	-	-	-	S.LP?m	II dlm 36, II dlm 37	t12, t13
IIdlm3	-	-	-	S.Esm	-	t-p 5, t-p 6
IIdvm1	x	x	23'	S.CmA	II dvm 40	t-ti 1, t-ti 2
IIdvm2	-	-	-	S.CmA	II dvm 41	t-ti 3
IIdvm3	x	x	23	-	-	t-cx 5
IIdvm4	x	x	26	PSL.Cm, S.CmP	II dvm 43	t-cx 6, t-cx 7
IIdvm5	x	x	27	SA.Cm, SA.Fm	II dvm 43	t-cx 8
IIdvm6	x	-	-	S.Trm	II cpm 53	t-tr 1
IIdvm7	-	-	-	-	II dvm 42	t-s 1
IIdvm8	-	-	-	-	II ism 44	t-s 8, t-s 7 ?
IIdvm9	-	-	-	A?.Pm	-	t-p 3
IItpm1	-	-	-	BA.Pm	II tpm 46a	t-p 4, t-p 20

Additional file 2: Homologisation of thoracic muscle nomenclatures used by several authors

"-" absent / "?" uncertain or no information

IItpm2	-	x	-	-	II tpm 47	t-p 7, t-p 8, t-p 9
IItpm3	x	-	-	SrA.Pm, Ax.Pml	II tpm 46b ?	t-p 10, t-p 11, t-p 18
IItpm4	x	x	28	-	-	t-p 12
IItpm5	-	-	-	-	-	t-p 15
IItpm6	x	x	31	-	II tpm 49	t-p 13
IItpm7	x	x	33	-	II tpm 48	-
IItpm8	x	x	32	Ax.PmS	-	t-p 14
IItpm9	x	x	29/30	-	-	t-p 16
IItpm10	x	x	34	-	II ppm 56	t-p 19
IItpm11	-	-	-	-	-	t-p 17
IItpm12	-	-	-	-	-	p 1
IItpm13	-	-	-	-	?	?
IIppm1	-	-	-	-	II im 65a	p 2
IIppm2	-	-	-	-	II pm 54a, b	p 3
IIspm1	-	-	-	-	II ppm 55	p-s 1
IIspm2	x	-	35	-	II zm 61a	p-s 2
IIspm3	-	-	-	-	-	p-s 6
IIspm4	-	-	-	-	-	p-s 7
IIspm5	-	-	-	-	-	p-s 3
IIspm6	-	-	-	-	-	p-s 9
IIspm7	-	-	-	-	-	p-s 5
IIspm8	-	-	-	-	-	p-ti(cx) 1
IIpcm1	x	x	21	-	-	p-ti(cx) 2, p-ti(cx) 3
IIpcm2	x	x	22	-	II cpm 51	p-cx 4, p-cx 6
IIpcm3	-	-	-	P.Cm	-	p-cx 5
IIpcm4	x	x	36	BA.Trm	II cpm 52	p-tr 2
IIpcm5	-	-	-	P.Trm	II cpm 50	p-tr 1
IIpcm6	x	x	39	Fm	-	s14, s16
IIvlm1	-	-	-	-	-	s 15

Additional file 2: Homologisation of thoracic muscle nomenclatures used by several authors

"-" absent / "?" uncertain or no information

IIvlm2	-	-	-	iFm	-	s 13
IIvlm3	-	-	-	-	II vlm 38	s 11
IIvlm4	-	-	-	-	-	s 12
IIvlm5	-	-	-	-	II vlm 39	p-s 13
IIvlm6	x	-	68	-	-	p-s 10
IIvlm7	x	x	41	F.CmA	-	s-cx 5
IIscm1	x	-	-	-	II bm 57	s-cx 3
IIscm2	x	-	-	-	II bm 60	s-cx 6
IIscm3	x	x	38	F.CmP	II bm 59	s-cx 2
IIscm4	-	-	-	-	II zm 61b	s-cx 4
IIscm5	-	-	-	-	-	s-tr1
IIscm6	x	x	40	-	II bm 58	s-cx 1
IIscm7	x	-	-	-	-	s-cx 7
Metathorax						
IIIdlm1	x	x	45	MT.m	III dlm 35	t 14
IIIdlm2	x	x	45'	S.LP?m	III dlm 36, III dlm 37	t 12, t13
IIIdlm3	-	-	-	-	III dvm 40	?
IIIdvm1	-	x	46'	S.Esm	III dvm 41	t-p 5, t-p 6
IIIdvm2	-	-	-	S.CmA	-	t-ti 1, t-ti 2
IIIdvm3	x	x	46	S.CmA	III dvm 43	t-ti 3
IIIdvm4	x	x	48	-	III dvm 43	t-cx 5
IIIdvm5	x	x	49	PSL.Cm, S.CmP	III cpm 53	t-cx 6, t-cx 7
IIIdvm6	x	-	46	SA.Cm, SA.Fm	III dvm 42	t-cx 8
IIIdvm7	-	-	-	-	III ism 44	t-tr 1
IIIdvm8	x	x	67	-	III tpm 46a	t-s 1
IIItpm1	-	-	-	A?.Pm	III tpm 47	t-p 3
IIItpm2	-	x	-	BA.Pm	III tpm 46b	t-p 4, t-p 20
IIItpm3	x	-	-	-	-	t-p 7, t-p 8

Additional file 2: Homologisation of thoracic muscle nomenclatures used by several authors

"-" absent / "?" uncertain or no information

IIItpm4	x	x	50	SrA.Pm, Ax.Pml	-	t-p 10, t-p 11, t-p 18
IIItpm5	-	-	-	-	III tpm 49	t-p 12
IIItpm6	x	x	53	-	III tpm 48	t-p 15
IIItpm7	x	x	55	-	-	t-p 13
IIItpm8	x	x	54	-	-	-
IIItpm9	x	x	51/52	Ax.PmS	III ppm 56	t-p 14
IIItpm10	x	x	56	-	-	t-p 16
IIItpm11	-	-	-	-	-	t-p 19
IIItpm12	-	-	-	-	-	t-p 17
IIItpm13	-	-	-	-	?	?
IIIppm1	-	-	-	-	III im 65a	p 1
IIIppm2	-	-	-	-	III ppm 54a, b	p 2
IIIspm1	-	-	-	-	III ppm 55	p 3
IIIspm2	x	-	57	-	III zm 61	p-s 1
IIIspm3	-	-	-	-	-	p-s 7
IIIspm4	-	-	-	-	-	p-s 9
IIIspm5	-	-	-	-		p-s 5
IIIspm6	-	-	-	-	?	?
IIIpcm1	x	x	43	-	-	p-ti(cx) 1
IIIpcm2	x	x	44	-	III cpm 51	p-ti(cx) 2, p-ti(cx) 3
IIIpcm3	-	-	-	-	-	p-cx 4, p-cx 6
IIIpcm4	x	x	58	P.Cm	III cpm 52	p-cx 5
IIIpcm5	-	-	-	BA.Trm	III cpm 50	p-tr 2
IIIpcm6	x	x	62	P.Trm	-	p-tr 1
IIIpcm7	-	-	-	-	-	p-cx 8
IIIvlm1	-	-	-	Fm	-	s 14, s16
IIIvlm2	x	x	65	-	III vlm 64	s 20
IIIvlm3	x	x	66	-	-	s 12
IIIscm1	x	-	-	F.CmA	III bm 57	s-cx 5

Additional file 2: Homologisation of thoracic muscle nomenclatures used by several authors

"-" absent / "?" uncertain or no information

IIIscm2	x	-	-	-	III bm 60	s-cx 3
IIIscm3	x	x	61	-	III bm 59	s-cx 6
IIIscm4	-	-	-	F.CmP	-	s-cx 2
IIIscm5	-	-	-	-	-	s-cx 4
IIIscm6	x	x	63	-	III cpm 58	s-tr 1
