# Supplementary material for: The thorax musculature of Anisoptera (Insecta: Odonata) nymphs and its evolutionary relevance
Source: BMC Evol Biol. 2013 Nov 1;13:237. doi: 10.1186/1471-2148-13-237 (PMC4228402; doi:10.1186/1471-2148-13-237)
Supplement: Additional file 3 — Thorax of Sympetrum vulgatum. A. Cross section of the pterothorax B. Sagital section C. Horizontal section (dorsal view). Cd - coxal disc, dvm - dorso-vetral musculature, Gt - gut, pcm - pleuro-coxal musculature, Pl - pleura, scm - sterno-coxal musculature, St - sternum, TAp - tergal apophysis, tpm – tergopleural musculature, Tr - trachee, vlm – ventral longitudinal musculature, WB - wing buds. [file 1471-2148-13-237-S3.pdf]

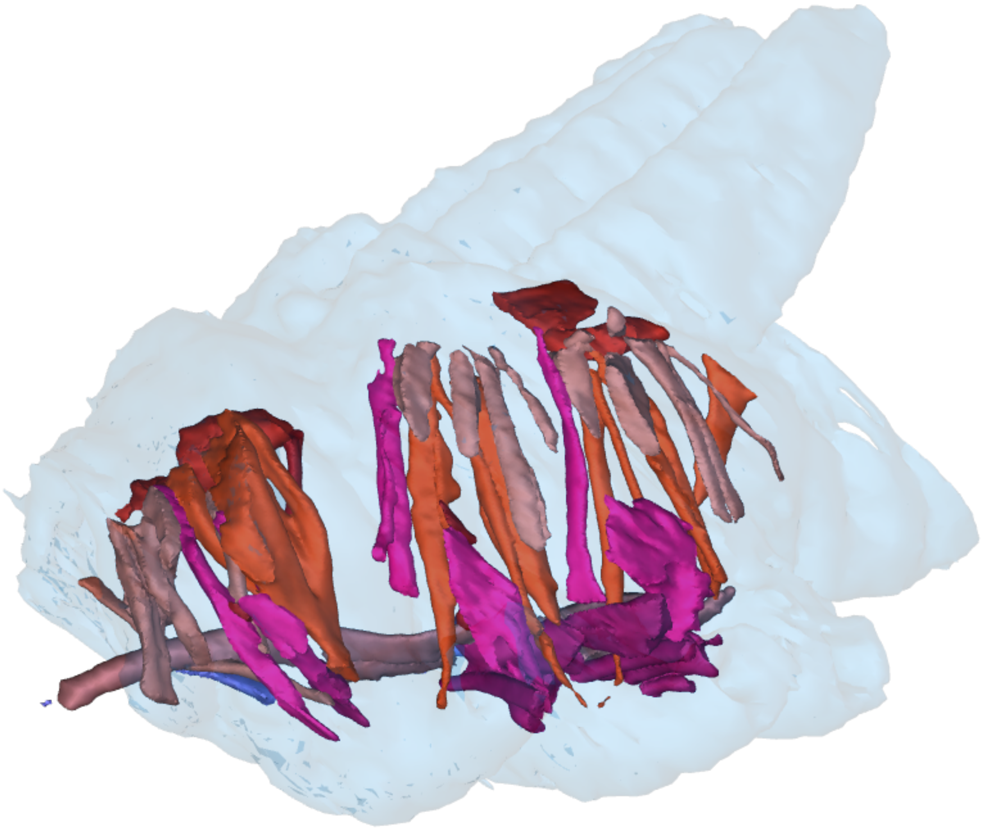

Additional file 3 -model of thoracic musculature of *Sympetrum vulgatum* nymph; reconstructed from SRµCT data showing transparent cuticle and muscles grouped as dorsal longitudinal (dlm), ventral longitudinal (vlm), dorso-ventral (dvm), tergo-pleural (tpm), sterno-pleural (spm), sterno-coxal (scm) and pleuro-coxal (pcm) muscles. After clicking the figure the model can be manipulated.

lateral	dorsal	ventral
pcm	scm	spm
dvm	dlm - vlm	tpm
