# Supplementary material for: The thorax musculature of Anisoptera (Insecta: Odonata) nymphs and its evolutionary relevance
Source: BMC Evol Biol. 2013 Nov 1;13:237. doi: 10.1186/1471-2148-13-237 (PMC4228402; doi:10.1186/1471-2148-13-237)
Supplement: Additional file 4 — Model of thoracic musculature of Sympetrum vulgatum nymph; reconstructed from SRμCT data showing transparent cuticle and muscles grouped as dorsal longitudinal (dlm), ventral longitudinal (vlm), dorso-ventral (dvm), tergopleural (tpm), sterno-pleural (spm), sterno-coxal (scm) and pleuro-coxal (pcm) muscles. After clicking the figure the model can be manipulated. [file 1471-2148-13-237-S4.pdf]

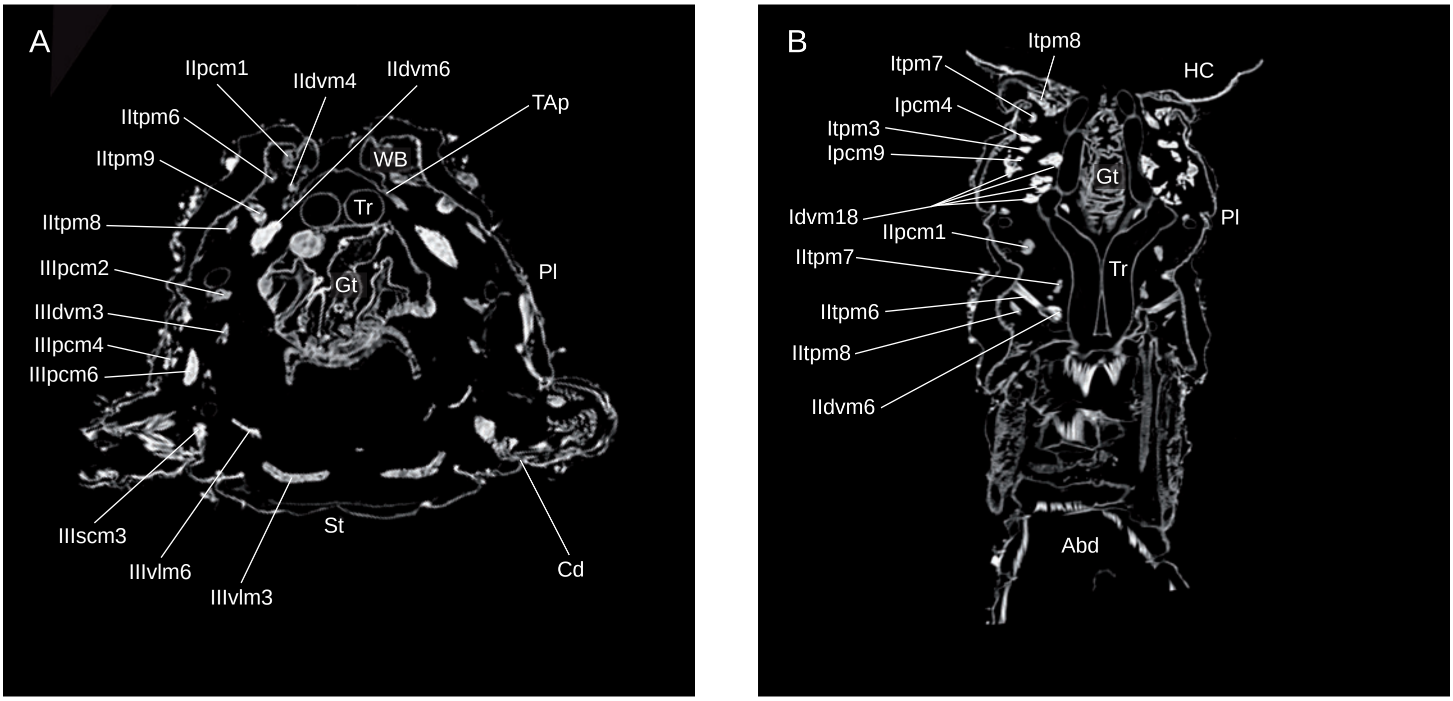

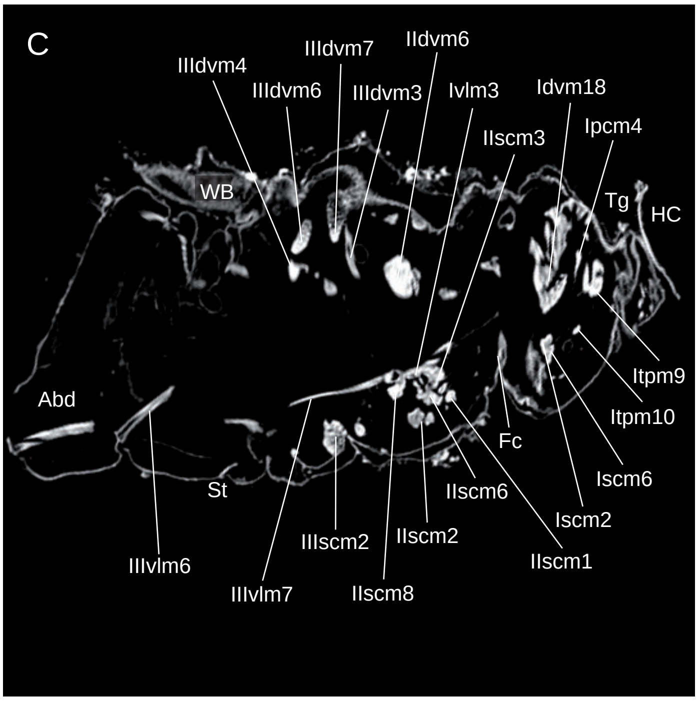

Additional file 4: Thorax of *Sympetrum vulgatum* A. Cross section of the pterothorax B. Sagital section C. Horizontal section (dorsal view).

Cd - coxal disc, dvm - dorso-vetral musculature, Gt - gut, pcm - pleuro-coxal musculature, Pl - pleura, scm - sterno-coxal musculature, St - sternum, TAp - tergal apophysis, tpm - tergo-pleural musculature, Tr - trachee, vlm - ventral longitudinal musculature, WB - wing buds.
